# DIET IN DERMATOLOGY: PRESENT PERSPECTIVES

**DOI:** 10.4103/0019-5154.70662

**Published:** 2010

**Authors:** K H Basavaraj, C Seemanthini, R Rashmi

**Affiliations:** *From the Department of Dermatology, JSS Medical College, Mysore - 570 015, India*

**Keywords:** *Dietary antioxidants*, *nutritional deficiency*, *skin immune system*

## Abstract

Many nutrients are essential for life, and an adequate amount of nutrients in the diet is necessary for providing energy, building and maintaining body organs, and for various metabolic processes. The role of food in the induction of various skin disorders and skin diseases leading to nutritional deficiencies is well known. The photo-protective potential of antioxidants, the effects of micronutrient supplementation on the skin immune system, and the modulating effects of fatty acids on skin disorders are well documented. Skin diseases due to nutritional deficiencies, the dietary role in skin immunity and various skin diseases, and the role of antioxidants and other supplements in skin health have been reviewed.

## Introduction

Nutrients are the chemical substances found in food. Many nutrients are essential for life, and an adequate amount of nutrients in the diet is necessary for providing energy, building and maintaining body organs, and for various metabolic processes. The skin (epidermis and dermis) functions normally when adequate nutrition is provided. For example, deficiency of essential fatty acids (EFA) is shown to increase epidermal permeability and transepidermal water loss.[1] Any dietary imbalance in the form of nutritional deficiency, specific nutrient inadequacy or excess and toxic components can disturb the equilibrium of the skin. Deficiencies of several vitamins, minerals, and fatty acids have clear cutaneous manifestations.[1–4] Skin diseases may lead to metabolic imbalances and cause nutritional deficiencies. The demand for nutrients in skin is altered under stress conditions. Excessive inflammation of the skin is known to increase the requirements of specific nutrients like folic acid and protein.[1] The photo-protective potential of antioxidants,[5] the effects of micronutrient supplementation on the skin immune system,[6] and the modulating effects of fatty acids on skin disorders,[3] have been the subject of a considerable number of studies.

## Nutritional Deficiency

**Figure 1:**
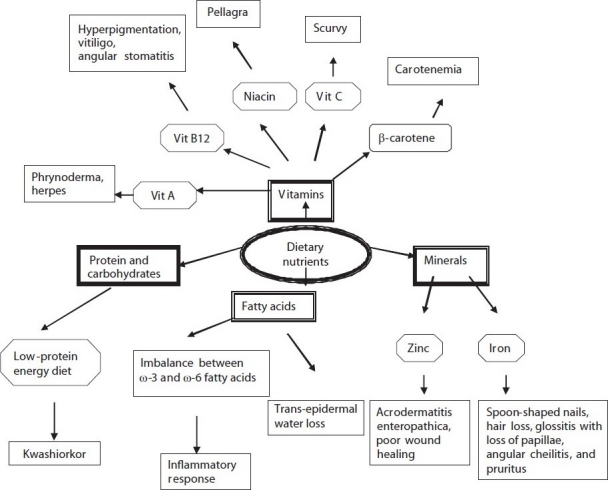
Dietary nutrients involved in various dermatologic conditions

Skin disorders have long been associated with nutritional deficiencies [Figure 1]. Earlier nutritional deficiency diseases were assumed to be limited to the underdeveloped and developing countries. In recent years nutritional deficiency diseases have been reported in developed countries.[7,8] Nutritional deficiencies can be due to inadequate intake, abnormal absorption or improper utilization.

Kwashiorkor is the edematous form of protein energy malnutrition. Evidence has been presented implicating aflatoxins, free oxygen radicals, leukotrienes, zinc deficiency, and essential fatty acid deficiency.[7]

Vitamin A is essential for the maintenance of differentiated epithelia. Deficiency of vitamin A results in hyperkeratinization with reduced number of sebaceous glands and blockage of sweat glands.[1] Hypovitaminosis A also affects the skin by causing xerosis, generalized hyperpigmentation, and sparse and fragile hair. Plugging of the follicular openings with spiny horns is one of the classic signs of vitamin A deficiency as in phrynoderma. Deficiency of other factors, such as Vitamin B, C, and E, calories, and essential fatty acids, have been incriminated in phrynoderma.[8]

Cutaneous manifestations associated with vitamin B12 deficiency are skin hyperpigmentation, vitiligo, angular stomatitis, and hair changes. Malabsorption is the most common cause of vitamin B12 deficiency. A detailed history of food and dietary habits has been suggested to evaluate skin lesions.[9]

Classic pellagra is a nutritional disease characterized by the combined deficiency of the essential amino acid tryptophan and the vitamin niacin.[2] Other factors, such as, mycotoxins, excessive dietary leucine intake, estrogens and progesterone, chronic alcoholism, and various medications, might also lead to the development of pellagra.[2,10,11]

Vitamin C is a cofactor for procollagen proline / lysine hydroxylase, and therefore, important in the synthesis of the collagen and extracellular matrix.[4,12] Vitamin C also aids in iron absorption and increases the conversion of cholesterol to bile acid and increases the bioavailability of selenium.[13] Scurvy is a deficiency disease of ascorbic acid manifested in the decreased production and increased fragility of collagen. Dermatologic signs that appear early in the disease include petechiae, echymoses, corkscrew or swan-neck hairs, follicular hyperkeratosis, and perifollicular hemorrhage.[12]

The cutaneous manifestations of zinc deficiency are weeping dermatitis, secondary infection, poor wound healing, excessively fragile hair and sparse or no scalp and pubic hair. Dermatitis, alopecia, and nail defects are also associated with zinc deficiency.[4] Acrodermatitis enteropathica develops in a zinc-deficient patient and a combined nutritional deficiency of zinc, EFAs, albumin and amino acids may result in acrodermatitis enteropathica.[14] The activities of lysyl oxidases that initiate the cross-linking of collagen and elastin decline with copper deficiency.[15] Chronic iron deficiency has resulted in spoon shaped nails (koilonychia), hair loss, glossitis with loss of papillae, angular cheilitis, and pruritus.[4]

**Figure 2:**
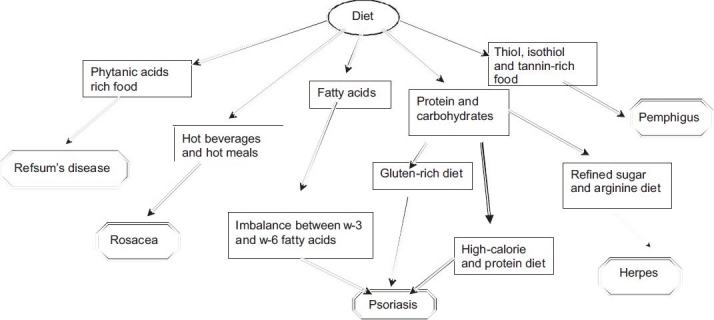
Diets known to precipitate various skin diseases

Excess of various nutrients can also result in certain diseases. Carotenemia is caused by excessive intake of carotene-rich food such as oranges and carrots.[16] Xanthelasmas may be associated with hyperlipidemia.[17] Phytanic acid is found in food stuffs like dairy products, meat, and fish,[18] [Figure 2] and impaired oxidation leads to Refsum’s disease causing a rough scaly thickening over the extremities.

Pemphigus is an autoimmune disease of the skin and mucous membranes that causes vesicles (blisters), bullae, and raw sores. The role of thiol- and tannin-rich foods in exacerbating pemphigus have been reviewed by Tur E and Brenner S [Figure 2].[19]

### Immunological aspects of diet

The skin is one of the target organs that is most often involved in food hypersensitivity reactions, as in atopic dermatitis, urticaria, and dermatitis herpetiformis. An immune mechanism is involved in the pathogenesis of these diet-related skin disorders.[20,21] Dermatitis herpetiformis is a well-known gluten intolerance disease and is controlled by a gluten-free diet.[21] Symptoms of atopic dermatitis and urticaria have been provoked in patients consuming foods such as, egg, milk, peanuts, tree nuts, soy, wheat, fish, and shellfish.[20]

The monohydroxy acids derived from polyunsaturated fatty acids got from dietary vegetable oil exhibit anti-inflammatory properties *in vitro*. Thus, supplementation of diets with appropriate purified vegetable oils, fish oil, or both may generate local cutaneous anti-inflammatory metabolites.[3]

Vitamin E has also been seen to decrease prostaglandin E2 production, as a result of which the T-cell proliferation and function may be enhanced. Vitamins have been reported to induce increased production of natural killer cells and to enhance their activity, as also to increase interleukin-2 production, and are known to stimulate humoral immune responses.[22]

Zinc deficiency is associated with delayed wound healing. Lim *et al*, have hypothesized the involvement of dietary zinc in activating the nuclear factor-kappa B (NFĸB), expression of proinflammatory cytokines (interleukin-1b and tumor necrosis factor-a), and in neutrophil infiltration during the early stage of cutaneous wound healing.[23] Turmeric, red pepper, cloves, ginger, cumin, anise, fennel, basil, rosemary, garlic, and pomegranate, can block NFĸB activation of inflammatory cytokines.[24]

### Role of dietary antioxidants and supplements

Antioxidant molecules in the skin, such as, glutathione, vitamin E, and vitamin C, interact with the reactive oxygen species (ROS) or their by-products to either eliminate them or to minimize their deleterious effects. Vitamin C is a water-soluble antioxidant, whereas, vitamin E is membrane-bound and capable of intercepting free radical-mediated chain reactions.[25] The observation that supplementation with vitamin E alone does not protect against sunburn has been explained by the fact that UV irradiation exposure has been previously seen to deplete vitamin E in the skin, as a result of oxidation. Vitamin E radicals had to be regenerated by other dietary antioxidants like vitamin C.[22]

Antioxidants are effective in reducing free radical damage of collagen and elastin, the fibers that support the skin structure, and in preventing wrinkles and other signs of premature aging.[26]

Supplementation with β-carotene[27] and other carotenoids,[27–29] such as dietary tomato paste containing lycopene[30] protects against UV-light-induced erythema in humans. The initially reported anticarcinogenic potential of β-carotene was based on its specific capacity to quench singlet oxygen, scavenge oxy-radicals, and terminate free radical reactions. However, they are reported to exacerbate UV carcinogenesis under certain dietary conditions, by acting as pro-oxidants, at high oxygen pressure and under oxidative stress.[5]

Selenium is an antioxidant mineral responsible for tissue elasticity. It also acts to prevent cell damage by free radicals. Selenium is an essential constituent of the enzyme glutathione peroxide, which in the presence of reduced glutathione, breaks down, potentially damaging the reactive peroxides. Associated skin signs include hypopigmentation of the skin and hair and whitening of the nails.[4] It may play an important role in preventing skin cancer, as it can protect the skin from damage from excessive ultraviolet light. Dietary sources of selenium include wheat germ, seafood such as tuna and salmon, garlic, Brazil nuts, eggs, brown rice, and whole-wheat bread.

Zinc plays a role as an antioxidant in protecting sulfhydryl groups from oxidation and prevents superoxide and hydroxyl radical production by pro-oxidant metals, copper, and iron. Therefore, zinc deficiency may increase oxidative stress-induced tissue damage by decreasing the antioxidant functions.[23]

It has been seen that tea extracts have greater antioxidant activity than most vegetables and fruits, and may be more potent antioxidants than vitamin C, E or carotenoids. Resveratrol is a potent, naturally derived antioxidant that has been studied for its cancer preventive effects in skin.[25]

Curcumin, a polyphenolic compound, isolated from the rhizome of the plant *Curcuma longa*, has traditionally been used for pain and wound-healing. The anti-inflammatory, antioxidant, chemopreventive, and chemotherapeutic activities of curcumin are well-documented.[31] Curcumin promotes faster wound healing in rats by increasing collagen synthesis and cell proliferation, and by decreasing the ROS.[32]

### Diet in various skin diseases

A low-calorie and low-protein diet has been recommended in the treatment of psoriasis [Table 1]. The positive effect of the low-calorie / energy diet is thought to be secondary to the modifications in the polyunsaturated fatty acid metabolism, which in turn influences the eicosanoid profile, including prostaglandins and thromboxanes.[33] The improvement of psoriatic symptoms is due to the lowering of the overall protein intake, by limiting epithelial proliferation and decreasing polyamine levels.[34] Psoriasis is an inflammatory condition that appears to be aggravated by an inflammatory diet. An inflammatory diet may consist of a food allergen or a diet with imbalanced ω-6 and ω-3 fatty acids.[35] Many psoriatic patients show increased sensitivity to gluten and their symptoms improve on a gluten-free diet.[34,35] A vegetarian-based diet may put an individual at a risk of eating high amounts of vegetable oils and soy products, and low amounts of fish, which can tip the balance toward a pro-inflammatory state. Low serum calcium and zinc during pregnancy is known to cause pustular psoriasis. Supplementations of food containing calcium and zinc have been suggested in such situations.[36] Iodide can precipitate pustular psoriasis. Seafood and iodized salt are rich sources of iodine.[37] Some of the medications and treatments used for psoriasis are known to create nutritional deficiencies. For example methotrexate is known to result in folic acid deficiency. The amount of folic acid in the diet can be increased by eating broccoli and green leafy vegetables, dried beans and peas, grapefruit and orange juice, cantaloupe, liver, and other organ meats, and fortified cereals.[35]

**Table 1:** Dietary changes shown to be beneficial in skin diseases

Disease	Diet
Psoriasis	Low-calorie and protein diet
	Balance ω-3 and ω-6 diet
	Gluten-free diet
Herpes	Elimination of refined sugar and arginine-rich diet
	Vitamins, zinc, and iron supplementation
Scleroderma	Vitamin E supplementation
	Elimination of high-fiber diet
Acne	Low-glycemic diet
	Elimination of skim milk
Rosacea	Elimination of hot beverages and hot meals
Pemphigus	Elimination of thiol, isothiol, and tannin-rich food
Refsum’s disease	Elimination of phytate-rich food

Herpes is a viral infection of the skin. It has been reported that recurrence of herpetic lesions can occur due to ingestion of refined sugar or arginine-rich food. However, the importance of dietary arginine as a causative factor has not been investigated scientifically.[38] A study suggested that a mix of nutrients, such as those found in fruits and vegetables, act together to maintain immune health, rather than individual dietary intakes of vitamins A, B6, C, and E, and of folic acid, zinc, and iron.[39]

Scleroderma is an autoimmune disease of the connective tissue, characterized by fibrosis and thickening of various tissues. Avoidance of high-fiber diet is advised to patients with scleroderma.[40] Improvement in the skin of scleroderma on vitamin E supplementation has been reported.[41]

Clement *et al*., in their studies have shown a positive association between the intake of skim milk and acne.[42] Robyn N Smith *et al*., have suggested that nutrition-related lifestyle factors may play a role in the pathogenesis of acne.[43] The role of chocolate and other dietary factors in acne development has also been reported.[44]

Childhood Vitiligo has been related to malnutrition and intake of junk food.[45] Consumption of coffee, tea, other hot drinks, tobacco, alcoholic beverages, spicy foods is known to precipitate rosacea.[36] Foods such as chocolate, cheese, coffee, yogurt, and some Japanese foods such as glutinous rice cake, soy sauce, and fermented soybeans are reported to play an important role in unpredictable, irregular aggravation of skin lesions in patients with atopic dermatitis.[46] Figure 2 depicts diets that are involved in precipitating different skin diseases.

## Conclusion

The association between skin disorders and nutritional deficiencies is well established. Relation between health and food has gained interest in recent years. Dermatologic conditions linked with nutrition can range from nutritional deficiencies, excess nutrients or metabolic disorders. Dietary modifications, although based on anecdotal reports or theoretical grounds, might help prevent recurrences of many skin diseases. *In vitro* studies and animal models have given us some insight in understanding the role of nutrients in skin diseases. However, there is a gap in the understanding on how combinations of nutrients, as they appear in the diet and when they are taken as multiple supplements, work *in vivo*. Further studies are required to fulfill this gap. The effective dosage and toxicity of nutritional supplements need to be defined.
